# A Model for Surface Defect Detection of Industrial Products Based on Attention Augmentation

**DOI:** 10.1155/2022/9577096

**Published:** 2022-05-14

**Authors:** Gang Li, Rui Shao, Honglin Wan, Mingle Zhou, Min Li

**Affiliations:** ^1^Shandong Computer Science Center, Qilu University of Technology (Shandong Academy of Sciences), Jinan 250014, China; ^2^School of Physical and Electronic Sciences, Shandong Normal University, Jinan 250014, China

## Abstract

Detecting product surface defects is an important issue in industrial scenarios. In the actual scene, the shooting angle and the distance between the industrial camera and the shooting object often vary, which results in a large variation in the scale and angle. In addition, high-speed cameras are prone to motion blur, which further deteriorates the defect detection results. In order to solve the above problems, this study proposes a surface defect detection model for industrial products based on attention enhancement. The network takes advantage of the lower-level and higher-resolution feature map from the backbone to improve Path Aggregation Network (PANet) in object detection. This study makes full use of multihead self-attention (MHSA), an independent attention block for enhancing the backbone network, which has made considerable progress for practical application in industry and further improvement of the surface defect detection. Moreover, some tricks have been adopted that can improve the detection performance, such as data augmentation, grayscale filling, and channel conversion of input images. Experiments in this study on internal datasets and four public datasets demonstrate that our model has achieved good performance in industrial scenarios. On the internal dataset, the mAP@.5 result of our model is 98.52%. In the RSDDs dataset, the model in this study achieves 86.74%. In the BSData dataset, the model reaches 82.00%. Meanwhile, it achieves 81.09% and 74.67% on the NRSD-MN and NEU-DET datasets, respectively. This study has demonstrated the effectiveness and certain generalization ability of the model from internal datasets and public datasets.

## 1. Introduction

**Figure 1:**
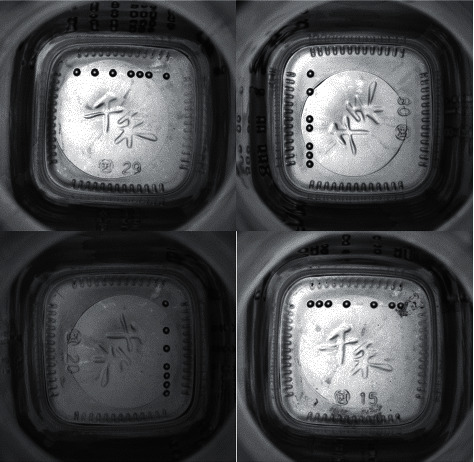
The mold points at the bottom of the glass bottle.

The detection of industrial products has always received a great deal of attention in computer vision. Most traditional methods rely on manual parameter setting, which is not conducive to higher detection accuracy and speed. Object detection based on convolutional neural networks has significantly progressed in recent years. Some well-known benchmark datasets, including MS COCO [1] and PASCAL VOC [2], have promoted the development of object detection applications. However, most of the object detection tasks are designed for images of natural scenes. There are three problems caused by these models for object detection in industrial scenes. First, with large variations of orientation and position during the shooting process with the industrial camera, the shape of the object significantly changes. Second, the images are captured at high speed, thus producing blurred objects. Third, images captured by industrial cameras often contain cluttered background because the targeted area is larger than the object area. For instance, a mirror image of the object produced by the object's background has interfered with the detection of the object, as shown in Figure 1. The above detection in industrial scenes is a challenging task. This study presents a targeted detection network in industrial settings. In this study, we come up with an attention-augmented object detection network, which improves the surface defect detection of existing industrial products. The network contains three key components, including the backbone, neck, and head. Referring to the backbone, it is composed of five convolutional groups and an independent attention block. Additionally, more feature information can be attained with the help of augmentation with independent attention blocks, which serves as an innovation for the backbone. In the neck, the SPP [3] structure and the PANet [4] structure are used to make the network more suitable for the detection of industrial defects. An FDS Block adopted in this study (fast downsampling block) is designed to quickly downsample the high-resolution feature in the backbone to concatenate well with the feature information in the neck.

The contributions of this study are listed as follows:

1. This study aggregates image feature information of lower level and high resolution in the backbone network into PANet, making the model collect richer feature information and improve feature extraction capabilities.
2. This study integrates the multihead self-attention (MHSA) mechanism into the detection network's backbone, which can advance the feature extraction ability and pay more attention to information.
3. This study provides a fast downsampling block (FDS Block) for the high-resolution images of the backbone network that quickly reduces the resolution and simultaneously increases the number of feature map channels. Based on that, high-resolution images in the backbone can be used to connect information with the highest-level, lowest-resolution feature maps in PANet as soon as possible.
4. We validate the effectiveness of our module through extensive ablation studies.
5. We propose a surface defect detection method for industrial products based on attention augmentation, which can perform well in industrial scenarios.

The structure of this study is organized as follows. Related works and the proposed method are described in detail, respectively, in Sections 2 and 3. Sections 4 and 5 tell the experimental results and conclusions of the study.

## 2. Related Works

### 2.1. Convolutional Neural Network

Since AlexNet [5] won the ImageNet competition in 2012, more and more convolutional neural networks have been proposed. The VGG [6] network won second place in the 2014 ILSVRC competition. On the basis of AlexNet, it has been greatly improved, that is to say, multiple smaller convolution kernels can replace the receptive field of large convolution kernels. ResNet [7] has shown that a network with residual blocks can expand the network depth to 101 layers. ResNeXt [8] proposed a separable convolution between the depths of the common convolution kernels, which makes a balance between the two strategies by controlling the number of groups. DenseNet [9] widened the network structure. DarkNet53 [10] and CSPDarkNet53 [11] are also proposed as popular methods.

### 2.2. Generic Object Detector

Modern detectors usually consist of two stages, a backbone pretrained on ImageNet and a head for predicting object classes and bounding boxes. The most representative two-stage object detector is the R-CNN [12] family, including Fast R-CNN [13], Faster R-CNN [14], and R-FCN [15]. The most representative one-stage object detectors are the YOLO family, involving YOLOv1 [16], YOLOv2 [17], YOLOv3 [10], and YOLOv4 [11]. At the same time, SSD [18] and RetinaNet [19] are one-stage object detectors. In recent years, anchor-free object detectors have developed. Such detectors include CenterNet [20], RepPoints [21], FCOS [22], and so on. Recently, object detectors have often constructed some layers (necks) between the head and the backbone, and these layers usually collect feature maps at different stages. The neck can generally cover multiple bottom-up paths and multiple top-down paths. Networks with this mechanism include the feature pyramid network (FPN) [23] and Path Aggregation Network (PANet).

### 2.3. Object Detection Effective Strategies

Data augmentation expands the dataset and makes the model more robust among datasets with different environments. Well-known data augmentation methods include MixUp [24], CutMix [25], and Mosaic [11]. MixUp randomly selects two samples from the training image for random weighted summation, which is in line with the labels of the samples. Unlike occlusion, which typically uses a zero-pixel “black cloth” to occlude an image, CutMix resorts to another image area to cover the occlusion. Mosaic is an improved version of CutMix, stitching the four images to greatly enrich the background of the detected object. Other data augmentation methods include DropBlock [26], class label smoothing [11], Cross mini-Batch Normalization (CmBN) [11], CIoU loss [27], DIoU loss [27], and mesh sensitivity elimination [11]. With multiple anchors, mesh sensitivity elimination will obtain a single ground truth, cosine annealing schedule, optimal hyperparameters, and random training shape. BOS (Bag of Specials), such as Mish activation function [11], Cross-Stage Partial Connection (CSP [28]), SPP, and PANet, can significantly heighten the accuracy of object detection with only a small increase in inference cost.

### 2.4. Attention Mechanism

In response to the defects of Seq2Seq [29], Bahdanau [30] proposed an attention mechanism to achieve a soft distinction and provide some visual effects of attention. Luong et al. [31] have put forward two improved versions of attention, such as global attention and local attention. Ahmed et al. [32] came up with a novel network structure transformer, which contains an attention mechanism called self-attention. Liu et al. [33] presented the gated multilingual attention (GMLATT) framework to solve such problems as data sparseness and monolingual ambiguity, using multilingual information combined with the attention mechanism to complete the task. Since the attention mechanism extracted key features by the weighted calculation of all local features and ignored the strong correlation between local features, there was robust information redundancy between features. In order to solve this problem, researchers from Meitu Cloud Vision and the Institute of Automation, Chinese Academy of Sciences, drew on the idea of PCA (principal component analysis) and proposed a self-attention model [34] that introduced the interactive perception of local features and combined them with the model embedded in a CNN network. The algorithm performs extremely well on behavior classification of multiple academic datasets and Meitu's internal industrial video dataset. Google released BoTNet [35], replacing the bottleneck of the fourth block in ResNet with the MHSA (multihead self-attention) module and forming a new module named Bottleneck Transformer (BoT). The final network structure, including blocks like BoT, is called BoTNet.

### 2.5. Industrial Defects

Zhang and Song [36] proposed a segmentation network to improve NRSD segmentation, which applied an attention mechanism to optimize the extracted information and performed well for both artificial and natural NRSDs. Niu and Song [37] proposed an unsupervised stereo saliency detection method based on a binocular line scanning system, which can simultaneously obtain high-precision image and contour information. He et al. [38] proposed a novel defect detection system based on deep learning and focused on steel plate defect detection, which uses a multilevel feature fusion network (MFN) to focus on multilevel features. Wu et al. [39] developed a more flexible deep learning method for industrial defect detection, and the author proposed a unified framework for detecting industrial products or flat surface defects. Xu et al. [40] established a defect detection network (D4Net) to detect deformed defects in a given image and its corresponding reference image.

## 3. Method

### 3.1. Network Overview

**Figure 2:**
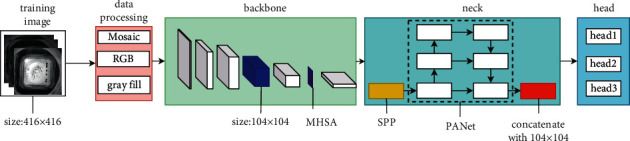
An overview of the detection process. Among them, the purple feature blocks in the backbone are attention enhancement structures.

The proposed model includes three parts: backbone, neck, and head. The backbone covers CSPDarknet53 and an attention-enhancing structure (purple block in the backbone in Figure 2). The neck part applies the PANet as the main part. In addition, this study decreases the resolution of the information on the 104 × 104 feature map (blue block in Figure 2 backbone) and fuses it with the feature map in PANet to form the red block in Figure 2 neck, which is input to the third detection head. This study adopts a series of strategies, i.e., data augmentation, grayscale filling, and automatic conversion of images to three-channel RGB, making the model more robust.

### 3.2. Structure

**Figure 3:**
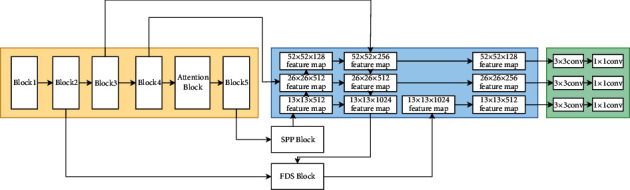
In the model structure diagram of this study, the yellow background is the backbone, the blue background is the neck, and the green background is the head.

In Figure 3, this study gives the most detailed explanation of the model, which is divided into two parts, A and B. Part A explains the backbone of the model, and Part B interprets the neck, SPP Block (spatial pyramid pooling block), and the FDS Block (fast downsampling block) that are connected with the neck by the trunk part.

#### 3.2.1. Attention-Enhanced Backbone

**Figure 4:**
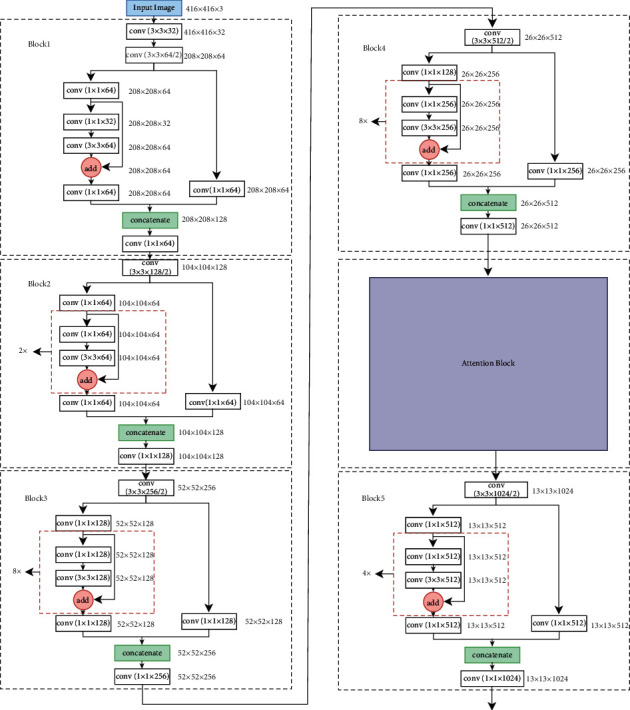
Details of blocks 1 to 5.

The backbone used in this study is CSPDarkNet53, as shown in Figure 3, and it is divided into five parts. Block1, Block2, Block3, Block4, and Block5 (details are shown in Figure 4). To meet the actual needs of industrial product defect detection and strengthen the feature extraction ability of the model to focus on more information, this study resorts to an attention enhancement strategy for the backbone.

**Figure 5:**
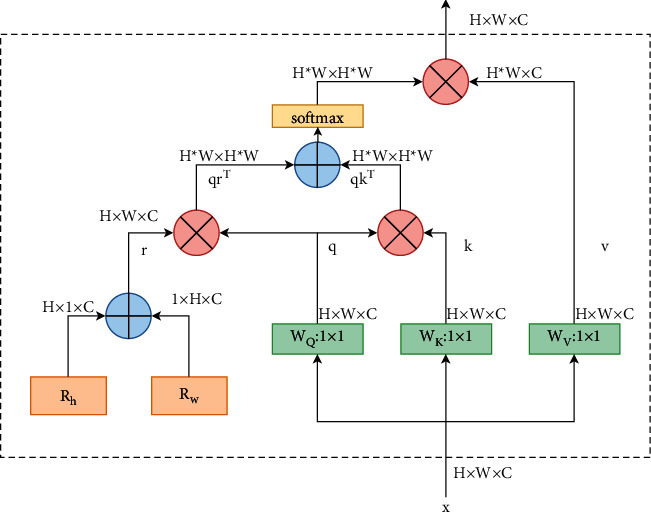
Attention block, where q, k, v and r refer to query, key, value, and position encoding, and Rh and Rw refer to height and height relative position encoding width.

This study uses MHSA to enhance CSPDarknet53. Transformers based on the self-attention mechanism are first applied in the NLP domain. In order to make the CNN backbone network with such characteristics, an effective method is to replace the spatial convolution layer in CNN with the MHSA proposed in transformer. As for our method, instead of replacing the convolution of the last residual layer with an MHSA layer like BoTNet, we choose the MHSA structure as the entire attention block before Block5 of the backbone network. In this study, the input resolution and output resolution of the attention block, the number of input channels, and the number of output channels are not changed. The attention block is shown in Figure 5.

In addition, this study adopts some detection enhancement strategies for the backbone, including the use of the Mish activation function and the Mosaic data enhancement method.

#### 3.2.2. Neck

**Figure 6:**
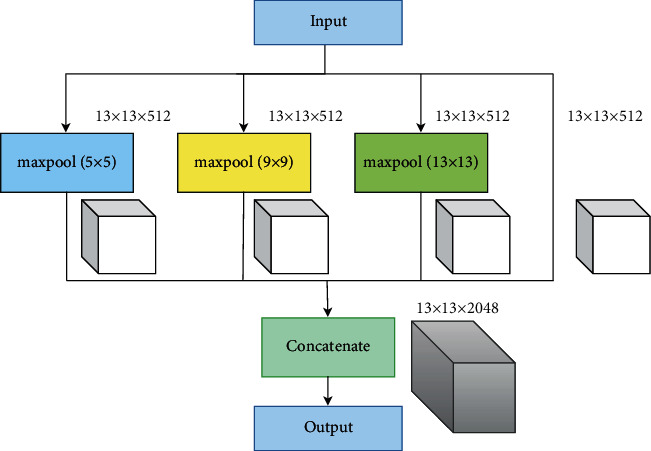
SPP structure.

As shown in Figure 3, the high-resolution feature map, SPP structure, and PANet structure in the backbone include the neck, making the network more suitable for detecting industrial product defects. The neck takes three feature maps of different output sizes from the backbone as input. The 13 × 13 size feature map passes into the SPP structure after three convolutions. Then, the SPP structure is formed by the max-pooling layer from three different kernel sizes of 5, 9, and 13. In addition, the SPP structure also includes one shortcut layer. After the SPP structure, the data go to the PANet structure. The 26 × 26 and 52 × 52 feature maps are directly fed into the PANet network after three convolutions. The SPP structure is shown in Figure 6.

**Figure 7:**
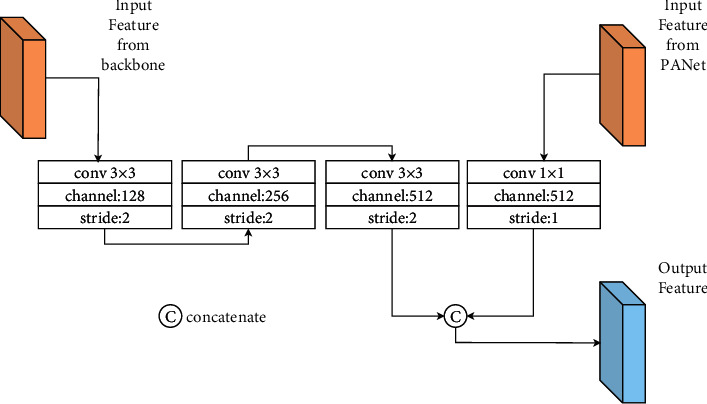
Structure of FDS block.

The PANet structure combines high-level, low-resolution feature maps with low-level, high-resolution feature maps bottom-up. Then, it connects the low-level, high-resolution feature maps with high-level, low-resolution feature maps from top to bottom. In this study, according to the experiments, the 104 × 104 feature map has the function of delivering rich information to the highest and lowest resolution layers. Therefore, the 104 × 104 × 128 feature map and the 13 × 13 × 1024 feature map in the PANet will be input to the FDS Block, and then, the 13 × 13 × 1024 feature map will be accordingly output. In doing so, we can not only collect rich feature information involved in the backbone, but also the output does not change the feature map size of PANet. The structure of the FDS Block structure is shown in Figure 7.

### 3.3. NMS of This Study

In this study, DIOU-NMS is used to obtain the most suitable detection box for each object, thereby improving the discriminative ability of the model in this study in the detection of surface defects of industrial products. The mathematical formula of DIOU-NMS is defined as follows:

(1) $$\begin{array}{c} S_{i}=\left\{\begin{array}{cc} S_{i}, & \text{IOU}-R\left(M,b_{i}\right)<N,\\ 0, & \text{IOU}-R\left(M,b_{i}\right)\geq N, \end{array}\right) \end{array}$$

where *b*_*i*_ is removed by considering both the IoU and the distance between the center points of the two boxes, *s*_*i*_ is the classification score, and *N* is the NMS threshold, where the mathematical formula for *R* is defined as follows:

(2) $$\begin{array}{c} R=\frac{\rho^{2}\left(b,b^{gt}\right)}{c^{2}}, \end{array}$$

where *ρ* is the distance, *b* and *b*^gt^ represent the two boxes, and *c* is the diagonal length of the smallest box containing the two boxes.

### 3.4. Loss Function

In this study, the expression of the loss function is as follows:

(3) $$\begin{array}{c} L=-\sum_{i=0}^{S^{2}}1_{ij}^{\text{obj}}\sum_{c\in N\text{classes}}\left[{\hat{p}}_{i}\left(c\right)\text{log}\left(p_{i}\left(c\right)+\left(1-{\hat{p}}_{i}\left(c\right)\right)\log\left(1-{\hat{p}}_{i}\left(c\right)\right)\right)\right]\\ +\lambda_{\text{coord}}\sum_{i=0}^{S^{2}}\sum_{j=0}^{B}1_{ij}^{\text{obj}}\left[{\left(x_{i}-{\hat{x}}_{i}\right)}^{2}+{\left(y_{i}-{\hat{y}}_{i}\right)}^{2}\right]+\\ \lambda_{\text{coord}}\sum_{i=0}^{S^{2}}\sum_{j=0}^{B}1_{ij}^{\text{obj}}\left[{\left(\sqrt{w_{i}}-\sqrt{{\hat{w}}_{i}}\right)}^{2}+{\left(\sqrt{h_{i}}-\sqrt{{\hat{h}}_{i}}\right)}^{2}\right]-\sum_{i=0}^{S^{2}}\sum_{j=0}^{B}1_{ij}^{\text{obj}}\left[{\hat{C}}_{i}\text{log}\left(C_{i}\right)+\left(1-{\hat{C}}_{i}\right)\text{log}\left(1-C_{i}\right)\right]\\ -\lambda_{\text{noobj}}\sum_{i=0}^{S^{2}}\sum_{j=0}^{B}1_{ij}^{\text{obj}}\left[{\hat{C}}_{i}\text{log}\left(C_{i}\right)+\left(1-{\hat{C}}_{i}\right)\text{log}\left(1-C_{i}\right)\right]. \end{array}$$

Overall, the first line of formulas is classification loss, the second and third lines of formulas are box regression loss, and the fourth and fifth lines of formulas are confidence loss.

## 4. Experimental Results

### 4.1. Experiment Description

#### 4.1.1. Dataset

**Figure 8:**
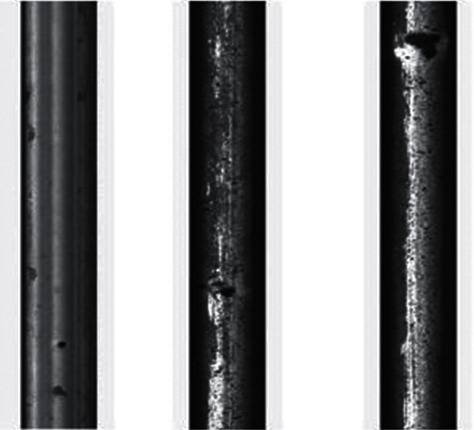
Example image of the RSDDs dataset.

**Figure 9:**
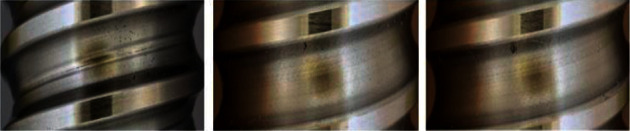
Example image of the BSData dataset.

**Figure 10:**
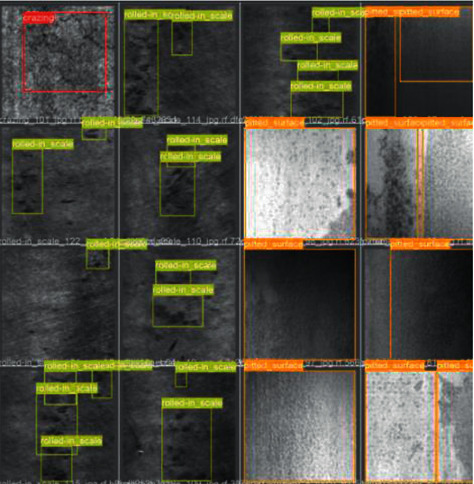
Example image of the NEU-DET dataset.

**Figure 11:**
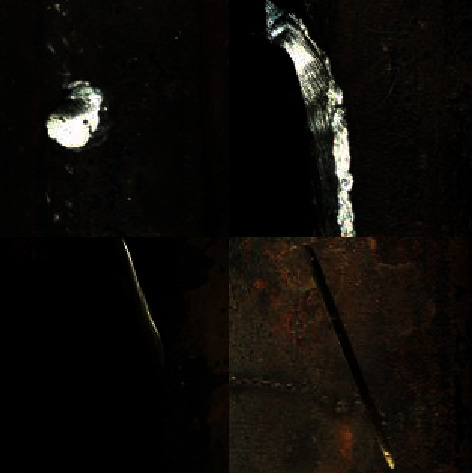
Example image of the NRSD-MN dataset.

Five datasets are exerted in this study, that is, four public datasets (RSDDs [41], BSData [42], NRSD-MN [36], and NEU-DET [38]) and one internal dataset for comparison with existing methods. The internal dataset is the image of the mold point at the bottom of the glass bottle, which is collected and saved from the actual production line with a CCD camera. The image resolution is fixed at 800 × 780. In addition, each dataset is divided into training, validation, and testing, with an amount ratio of 5 : 2 : 3. All object detection images refer to different colors to represent the corresponding types. RSDDs (Railway Surface Defects Dataset) contains two types of datasets. The first one is the type I captured from the fast lane, covering 67 challenging images. The second is a class II captured from normal/heavy-transport tracks, containing 128 challenging images. Each image from both datasets includes at least one defect with complex and noisy backgrounds. Referring to the experiments in this study, object detection is performed on the type I dataset with a resolution of 160 × 1000. Thirty-two images are selected in this study as the training set, 14 as the validation set during training, and 21 as the test set.  Figure 8 shows three examples of images and defects. The image size of BSData is 1130 × 460. This study chooses a subset of 394 defect images, of which 192 for training, 83 for training validation, and 119 for testing. Furthermore, Figure 9 shows some example pictures. The internal dataset of this study contains 481 training images, 207 validation images during training, and 295 testing images. Example images are shown in Figure 1. NEU-DET dataset is a defect classification data set. There are six types of defects from hot-rolled steel plates, including crazing, inclusion, patches, pitted surface, rolled-in scales, and scratches. The dataset has a total of 1800 images. This study selects 1260 images as the training set and 540 as the test set. Example images are shown in Figure 10. The NRSD-MN dataset contains 4101 images, including 3936 man-made NRSD images and 165 natural NRSD images. In this study, 4101 images are selected as our training and test sets, compared with the state-of-the-art algorithm. Among them, we take 2971 images as training set and 1130 images as test set. Example images are shown in Figure 11.

#### 4.1.2. Implementation

This study implements the model described in this study on PyTorch 1.9.0. The computing performance of industrial computers is strong or weak. In order to make the model have good detection ability on computers with different performances, this study trains and tests the model on GPUs with different performances. Ablation experiments are performed on NVIDIA RTXA6000 for the internal and RSDDs dataset, ablation experiments on NEU-DET and NRSD-MN datasets are performed on NVIDIA RTX 3080, and ablation experiments for the BSData dataset are performed on NVIDIA Tesla k80. In the model in this study, after training and testing images entered into the network, the resolution is unified to 416 × 416. The batch size is 8 on NVIDIA RTXA6000, 8 on NVIDIA RTX 3080, 4 on NVIDIA Tesla k80, and 2 on NVIDIA RTX 3060. This study has two parts during training, which are divided into the freeze training part and the unfreeze training part. Freeze training sees that the backbone is frozen and sees the unchanged feature extraction network. The initial learning rate becomes 0.001 during freeze training and 0.0001 after freeze training, and the learning rate decay is annealed cosine.

#### 4.1.3. Performance Metrics for Object Detection

The method proposed in this study provides defect localization and defect type classification for defect objects.

The IoU (intersection over union) measures the degree of overlap between two regions, which is the ratio of the overlapping area of the two regions to the total area (the overlapping part is only calculated once). As for the object detection task, the model output is considered to be correct till the IoU value of the rectangular box output by the model and the rectangular box manually marked is greater than a certain threshold.

Precision and recall can be expressed as follows:

(4) $$\begin{array}{c} \text{Precision}=\frac{TP}{TP+FP},\\ \text{Recall}=\frac{TP}{TP+FN}. \end{array}$$

As the name indicates, AP means the average precision. Simply put, it is the average of the precision values on the PR curve. For the PR curve, this study employs the integral to calculate it.

(5) $$\begin{array}{c} AP=\int_{0}^{1}p\left(r\right)\text{d}r. \end{array}$$

The mAP is a general model evaluation criterion in the field of object detection. The object detection in this study is used for detecting multiple objects. Therefore, this study can calculate the mAP.

The mAP can be expressed as follows:

(6) $$\begin{array}{c} mAP=\frac{\sum_{n=1}^{N}\int_{0}^{1}p\left(r\right)\text{d}r}{N}. \end{array}$$

### 4.2. Results

This study uses the test set of the internal dataset, the test set of the RSDDs dataset, the test set of the BSData dataset, the test of the NEU-DET dataset, and the test of the NRSD-MN dataset to evaluate our model and report mAP (using the metric mAP@.5 of the PASCAL VOC dataset).

**Figure 12:**
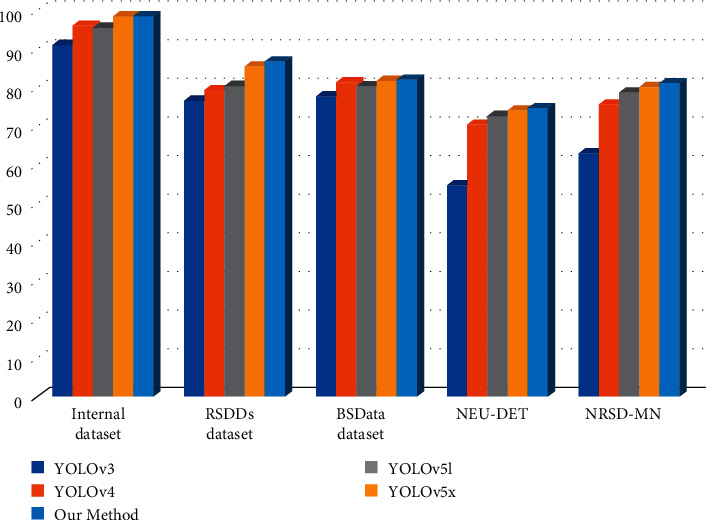
Comparison of mAP in five datasets.

In the end, this study achieves a good score of 98.52 on the internal dataset, which is higher than the state-of-the-art model yolov5x highest score of 98.45 on the internal dataset. Moreover, a good score of 86.74 on the RSDDs test dataset is performed, higher than the yolov5x highest score of 85.45 on the RSDDs dataset. It also gets a good score of 82.00 on the BSData test dataset, which is higher than the highest score of yolov5x on the BSData dataset of 81.79. Subsequently, in the NEU-DET dataset and the NRSD-MN dataset, our model achieved 74.67 and 81.09, respectively. As listed in Table 1, the scores of our model can compare their scores among YOLOV3, YOLOV4, YOLOv5l, and YOLOv5x algorithms.  Figure 12 shows the comparison of mAP in the five datasets.

The comparative experiments on the internal dataset in this study are performed on NVIDIA RTX 3060 with batch size set to 2. This study conducts freeze training for 100 epochs followed by 100 epochs after unfreezing. The comparative experiments in Table 1 demonstrate the detection ability of the model in the internal dataset.

The comparative experiments on the RSDDs dataset are conducted on NVIDIA RTX 3060 with batch size set to 2. This study makes freeze training for 100 epochs and continues training for 100 epochs after unfreezing. The comparative experiments in Table 1 show the detection ability of the model in the RSDDs dataset.

The comparative experiments on the BSData dataset are made on NVIDIA RTX 3060 with batch size set to 2. In order to verify the ability of the model and fit the data faster and better, this study only carries out freeze training for 50 epochs. Only 50 epochs will be continuously trained after unfreezing. The comparative experiments in Table 1 demonstrate the detection ability of the model in this study in the BSData dataset.

The comparative experiments on the NEU-DET dataset and the NRSD-MN dataset are made on NVIDIA RTX 3080 with batch size 8. In order to verify the ability of the model and fit the data faster and better, this study only carries out freeze training for 200 epochs. The comparative experiments in Table 1 demonstrate the detection ability of the model in this study in the NEU-DET dataset and the NRSD-MN dataset.

### 4.3. Ablation

#### 4.3.1. On the Internal Dataset

This study utilizes a test set of internal datasets (including images of glass bottle bottom mold points collected in actual industrial production) to evaluate our model and report mAP (including the metric mAP@.5 of the PASCAL VOC dataset).  Table 2 recites the scores of the models.

The ablation experiments in Table 2 exhibit the detection ability of each improved module of the proposed model in the internal dataset. With attempts to advance the use of higher-resolution images (832 × 832), the fitting ability does not meet expectations, followed by an increased amount of computation by two times. Although this study tries to use 104 × 104 feature information to add to P3 and pass the result to the detection head, the effect is far from expectations. Therefore, this study no longer makes an attempt on 832 × 832 images in the subsequent experiments nor does it add 104 × 104 feature information to P3 in the PANnet structure.

Effects of Attention Blocks. The addition of an attention block increases the amount of computation, but mAP improves very well. It can be seen from Table 2 that the CSPDarkNet53+ attention block performs well in object detection, with an increase of 1.45%. The introduction of attention block is worthwhile.

Effects of FDS Block. Using lower-layer, higher-resolution feature maps for fusion improves mAP and does not have a large impact on computation and inference speed. It also plays a certain role in detecting dense and large objects. As shown in Table 2, by adding 104 × 104 feature information to the P5 layer on PANet, mAP increases by 1%.

Aggregation Effect. This study lists the mAP of all the results of the ablation experiment. It is found that adding all innovation points to the model at the same time benefits the most, and mAP achieves the best results.

#### 4.3.2. On the RSDDs Dataset

This study uses the test set of the RSDDs dataset to evaluate our model and report mAP (using the metric mAP@.5 of the PASCAL VOC dataset).  Table 3 lists the scores of the models.

The ablation experiments in Table 3 demonstrate the detection ability of each improved module in the RSDDs dataset.

Impact of Attention Block. It can be seen from Table 3 that the CSPDarkNet53+ attention block performs well in object detection, with an increase of 1.33%. The introduction of attention block is worthwhile.

The Effect of FDS Block. As shown in Table 3, by adding 104 × 104 feature information to the P5 layer in PANet, mAP increases by 2.11%.

Aggregation Effect. This study enumerates the mAP of all the results of the ablation experiment. It is found that adding all innovation points to the model simultaneously benefits the most, and mAP gets the best results. As shown in Table 3, mAP increases by 7.47%.

#### 4.3.3. On the BSData Dataset

This study uses the test set of the BSData dataset to evaluate our model and report mAP (using the metric mAP@.5 of the PASCAL VOC dataset).  Table 4 lists the scores of the models.

The ablation experiments in Table 4 demonstrate the detection ability of each improved module of the model on the BSData dataset.

Effects of Attention Blocks. As Table 4 shows, the CSPDarkNet53+ attention block performs well in object detection, with an increase of 0.21%. So it is well worth making an introduction to the attention block.

Effects of FDS Block. As shown in Table 4, by adding 104 × 104 feature information to the P5 layer on PANet, mAP increases by 0.41%.

Aggregation Effect. This study shows the mAP of all the results of the ablation experiment. We find that adding all innovation points to the model at the same time behaves well, and mAP achieves the best results. As shown in Table 4, mAP has increased by 0.62%.

In order to test the fast fitting ability of the model, this study only performs freeze training for 50 epochs. In addition, 50 epochs will be carried out after thawing, which improves the detection effect. This study believes that the model will have better detection results in light of more sufficient computing conditions.

#### 4.3.4. On the NEU-DET Dataset

This study uses the test set of the NEU-DET dataset to evaluate our model and report mAP (using the metric mAP@.5 of the PASCAL VOC dataset).  Table 5 lists the scores of the models.

The ablation experiments in Table 5 demonstrate the detection ability of each improved module of the model on the NEU-DET dataset.

Effects of Attention Blocks. As Table 5 shows, the CSPDarkNet53+ attention block performs well in object detection, with an increase of 1.73%. So it is well worth making an introduction to the attention block.

Effects of FDS Block. As shown in Table 5, by adding 104 × 104 feature information to the P5 layer on PANet, mAP increases by 0.87%.

Aggregation Effect. This study shows the mAP of all the results of the ablation experiment. We find that adding all innovation points to the model at the same time behaves well, and mAP achieves the best results. As shown in Table 5, mAP has increased by 4.32%.

#### 4.3.5. On the NRSD-MN Dataset

This study uses the test set of the NRSD-MN dataset to evaluate our model and report mAP (using the metric mAP@.5 of the PASCAL VOC dataset).  Table 6 lists the scores of the models.

The ablation experiments in Table 6 demonstrate the detection ability of each improved module of the model on the NRSD-MN dataset.

Effects of Attention Blocks. As Table 6 shows, the CSPDarkNet53+ attention block performs well in object detection, with an increase of 2.51%. So it is well worth making an introduction to the attention block.

Effects of FDS Block. As shown in Table 6, by adding 104 × 104 feature information to the P5 layer on PANet, mAP increases by 1.05%.

Aggregation Effect. This study shows the mAP of all the results of the ablation experiment. We find that adding all innovation points to the model at the same time behaves well, and mAP achieves the best results. As shown in Table 6, mAP has increased by 5.69%.

## 5. Conclusions

This study proposes a surface defect object detector for industrial products, which is especially good at detecting product surface defects in industrial scenarios. Experiments on an internal dataset and four public datasets (RSDDs, BSData, NRSD-MN, and NEU-DET) have been carried out. Experiments show that the model in this study has good performance. This study argues that with rich computing resources, a longer training time can be used to allow the model to get better detection results. On the basis of previous research work, this study plans to further improve the object detection network structure to achieve better industrial detection performance in the future, including higher accuracy, faster test speed, and better prediction stability. At the same time, in the face of more difficult and deeper defect detection, on the one hand, this study intends to use camouflaged object detection to conduct experiments and research. On the other hand, it is committed to helping developers and researchers analyze and process scenes captured in industrial machine vision.

## Figures and Tables

**Table 1:** Comparison with state-of-the-art methods in three different datasets.

Methods	(Internal) mAP	(RSDDs) mAP	(BSData) mAP	(NEU-DET) mAP	(NRSD-MN) mAP
YOLOv3	91	76.60	77.63	54.7	63.09
YOLOv4	95.97	79.27	81.38	70.35	75.4
YOLOv5l	95.53	80.43	80.39	72.7	78.71
YOLOv5x	98.45	85.45	81.79	74.07	80.15
Our method	98.52	86.74	82.00	74.67	81.09

**Table 2:** Ablation studies on the internal dataset.

Number	Methods	mAP
A	CSPDarkNet53 + SPP + PANet	95.97
B	A + 832 × 832 resolution	95.33 (↓0.64)
C	A + 104 × 104 feature to P3	95.75(↓0.22)
D	A + FDS block	96.97 (↑1.00)
E	A + attention block	97.42 (↑1.45)
F	Our method (D + E)	98.52 (↑2.55)

**Table 3:** Ablation studies on the RSDDs dataset.

Number	Methods	mAP
A	CSPDarkNet53 + SPP + PANet	79.27
B	A + attention block	80.6 (↑1.33)
C	A + FDS block	81.38 (↑2.11)
D	Our method (B + C)	86.74 (↑7.47)

**Table 4:** Ablation experiments on the BSData dataset.

Number	Methods	mAP
A	CSPDarkNet53 + SPP + PANet	81.38
B	A + attention block	81.59 (↑0.21)
C	A + FDS block	81.79 (↑0.41)
D	Our method (B + C)	82.00 (↑0.62)

**Table 5:** Ablation experiments on the NEU-DET dataset.

Number	Methods	mAP
A	CSPDarkNet53 + SPP + PANet	70.35
B	A + attention block	72.08 (↑1.73)
C	A + FDS block	71.22 (↑0.87)
D	Our method (B + C)	74.67 (↑4.32)

**Table 6:** Ablation experiments on the NRSD-MN dataset.

Number	Methods	mAP
A	CSPDarkNet53 + SPP + PANet	75.4
B	A + attention block	77.91 (↑2.51)
C	A + FDS block	76.45 (↑1.05)
D	Our method (B + C)	81.09 (↑5.69)

## Data Availability

The dataset can be accessed upon request to the corresponding author.
